# Evaluating C-Reactive Protein in Drainage Fluid as a Predictive Biomarker for Clinically Relevant Pancreatic Fistulas Following Pancreaticoduodenectomy

**DOI:** 10.1245/s10434-026-19140-z

**Published:** 2026-02-07

**Authors:** Kenta Baba, Kenichiro Uemura, Tatsuaki Sumiyoshi, Ryuta Shintakuya, Kenjiro Okada, Takumi Harada, Shiro Oka, Yasutaka Ishii, Koji Arihiro, Yoshiaki Murakami, Shinya Takahashi

**Affiliations:** 1 Department of Surgery, Graduate School of Biomedical and Health Science, Hiroshima University, Hiroshima, Japan; 2 Department of Gastroenterology, Graduate School of Biomedical and Health Sciences, Hiroshima University, Hiroshima, Japan; 3 Department of Anatomical Pathology, Hiroshima University Hospital, Hiroshima, Japan; 4 Digestive Disease Center, Hiroshima Memorial Hospital, Hiroshima, Japan

**Keywords:** C-reactive protein, Drainage, Pancreatectomy, Pancreatic fistula, Postoperative complications

## Abstract

**Background:**

This study was conducted to determine the use of C-reactive protein (CRP) levels in drainage fluid as an alternative indicator of clinically relevant postoperative pancreatic fistulas (CR-POPFs) following pancreaticoduodenectomy (PD). Reportedly, serum CRP levels are associated with CR-POPFs. Drainage fluid following PD is used to diagnose a pancreatic fistula, removing the need for blood sampling. However, the significance of CRP in drainage fluid remains unclear.

**Methods:**

In this retrospective study, we reviewed consecutive patients who underwent PD at Hiroshima University between April 2014 and October 2021. Clinical factors for CR-POPFs were analyzed, and serum and drain CRP levels were measured during the first 4 days after surgery.

**Results:**

Among the 384 patients enrolled, 58 (15.1%) developed CR-POPFs. Serum and drain CRP levels on postoperative days (PODs) 2, 3, and 4 were significantly associated with CR-POPFs. Receiver operating characteristic analysis indicated that CRP levels on POD 4 most precisely predicted CR-POPFs, with area under the curve values of 0.820 and 0.803 for serum and drain CRP levels, respectively. Additionally, these two factors exhibited a strong positive correlation (*p*<0.001, *r*=0.814). Multivariate analysis revealed that drain CRP level ≥6.5 mg/dL on POD 4 independently predicted CR-POPFs (odds ratio 4.95; 95% confidence interval 2.27–10.81; *p*<0.001) with a negative predictive value of 95.6%.

**Conclusions:**

Drain CRP level on POD 4 is a strong predictive factor for CR-POPFs and may be an alternate criterion to serum CRP levels after PD.

Postoperative pancreatic fistulas (POPFs) after pancreaticoduodenectomy (PD) are relatively common and remain a major cause of morbidity and surgical mortality. The International Study Group of Pancreatic Surgery (ISGPS) Evidence Map of Pancreatic Surgery reported a postoperative mortality rate of 2% and a clinically relevant POPF (CR-POPF) rate of 23%.^1^ Therefore, early identification of patients at high risk of POPF is crucial.

Various studies have investigated the perioperative factors that influence POPF incidence.^2–8^ Drainage fluid following PD is used to diagnose a pancreatic fistula.^4^ Reportedly, the drain amylase level on postoperative day (POD) 1 suggests POPF development.^9,10^ Furthermore, a recent report demonstrated the predictive value of the kinetics between drain fluid amylase on PODs 1 and 3;^11^ however, few studies have examined drain biochemical markers other than amylase.

While amylase levels in drainage fluid are useful for early detection of pancreatic juice leakage, they may not reflect the inflammatory burden or clinical severity of the fistula. CR-POPFs (grades B and C), as defined by the ISGPS, are associated with infectious complications, prolonged drainage, or the need for intervention. In contrast to amylase, C-reactive protein (CRP) is a well-established marker of systemic inflammation and has been shown to correlate with the severity of postoperative complications. Serum CRP levels have been associated with CR-POPFs in several previous studies and may provide additional value in predicting not only the presence but also the impact of POPFs. Thus, CRP—especially in drain fluid—might serve as a complementary or alternative biomarker for assessing the clinical relevance of POPFs.

The level of CRP, an acute-phase protein, is increased in different inflammatory disorders and can be used to identify the causes of various diseases.^12^ Studies have shown that serum CRP levels are associated with postoperative infectious complications.^13–16^ Additionally, serum CRP levels correlate with CR-POPFs following PD.^3,6,16–26^ However, the significance of CRP levels in the drainage fluid after PD remains unclear. Therefore, this study aimed to determine the role of drain CRP levels as an alternative indicator of serum CRP levels in CR-POPFs after PD.

## Methods

### Study Design

This retrospective, comparative cohort study was conducted based on data obtained from institutional medical records. Although CRP levels in drain fluid were measured prospectively as part of routine care, the analysis and model construction were performed retrospectively. Consecutive patients who underwent PD with curative intent for biliary-pancreatic cancers and potentially malignant tumors (intraductal papillary mucinous neoplasms and neuroendocrine neoplasms) at the Department of Surgery, Hiroshima University Hospital, Hiroshima, Japan, between April 2014 and October 2021 were enrolled. Patients who underwent hepatopancreatoduodenectomy or total pancreatectomy and those with insufficient medical records were excluded. This study was approved by the institutional review board of Hiroshima University (E2023-0252) and conducted in accordance with the principles of the Declaration of Helsinki. Informed consent was obtained from all patients. This study used an intention-to-treat analysis.

### Patient Data Collection and Examination of Blood and Drainage Fluid Samples Following PD

Serum CRP levels and amylase and CRP levels from surgical drains placed alongside the pancreaticogastrostomy or pancreaticojejunostomy were prospectively measured daily for ≥4 days. Drain fluid samples were collected daily from POD 1 to POD 4, and both amylase and CRP levels were measured from the same sample using standard laboratory protocols. CRP measurement in drain fluid was performed using the same latex agglutination assay as for serum CRP, without any special modifications. Although reference ranges for CRP levels in drainage fluid are not standardized, these values were treated as continuous variables and analyzed using receiver operating characteristic (ROC) methods to determine optimal thresholds. Drain fluid appearance or quality was not systematically recorded in the institutional database during the study period and so was not included in the analysis. Data on clinical outcomes, patient characteristics, and pathological diagnoses were obtained. Data on patient characteristics, including age, sex, body mass index, diabetes, preoperative bile drainage, neoadjuvant chemotherapy, and the American Society of Anesthesiologists Physical Status (ASA-PS), were collected. Operative findings, including the type of pancreatic anastomosis after resection, vessel resection, pancreatic cancer, pancreatic texture, pancreatic duct diameter, estimated blood loss, and intraoperative blood cell transfusion, were recorded. Postoperative outcomes, including postoperative pancreatic fistula, postoperative complications according to the Clavien–Dindo classification,^27^ length of hospital stay, and 30-day mortality rate, were also collected.

### Definition of Morbidity and Mortality

POPF, delayed gastric emptying (DGE), and postpancreatectomy hemorrhage (PPH) are well-known major morbidities after PD. In this study cohort, the definitions of POPF,^4^ DGE,^28^ and PPH^29^ were based on the ISGPS criteria and graded as A, B, or C according to the clinical severity, with C being the most severe. Grade B and C pancreatic fistulas were defined as CR-POPF. Only grades B/C POPF, DGE, and PPH were considered in the analysis of the incidence of postoperative morbidity. Data on complications, such as abdominal abscess, anastomotic leakage, ascites, bile leakage, ileus, and postoperative hyperamylasemia (POH), were also collected. The incidence and severity of each morbidity were graded according to the Clavien–Dindo classification system.^27^ POH was defined as an elevation of serum amylase above the upper limit of normal (110 U/L) on POD 2. For comparative purposes, only grade ≥III complications were considered when analyzing morbidities. These morbidities were defined as adverse events that caused a deviation from the normal course on POD 60. Mortality was defined as the 30-day mortality during the initial hospital stay for surgery. The Pancreatic Fistula Risk Score (FRS) was calculated for all patients to compare the predictive performance of drain CRP with an established CR-POPF risk model.^7^ The original intraoperative blood loss–included FRS model was used in this study.

### Surgical Procedure and Postoperative Management

Regarding the surgical procedure, PD was performed via laparotomy. In all patients who underwent PD, duct-to-mucosa pancreaticogastrostomy or duct-to-mucosa pancreaticojejunostomy was performed using an internal stent.^30^ After pancreatic reconstruction, end-to-side hepaticojejunostomy and end-to-side duodeno- (or gastro-) jejunostomy were performed using the antecolic Roux-en Y technique. Two closed drains (Soft Pleats Drain; Sumitomo Bakelite Co., Tokyo, Japan) were placed in all patients, and pancreatogastrostomy (or pancreaticojejunostomy) and hepaticojejunostomy were performed during surgery.^6^ The indications for the proper management of surgical drains following PD have been previously described.^6,31,32^ The indications for drain removal were the serous color of the drain fluid and serum CRP levels <15.6 mg/dL on POD 4.^6^ The drains were to be removed by POD 5 when pancreatic fistula and bacterial contamination were absent.

### Statistical Analysis

Categorical clinicopathological variables were compared between the CR-POPF and non-CR-POPF groups using the Chi-squared test, whereas continuous clinicopathological variables were compared using the Wilcoxon two-sample test. Continuous data are presented as medians with 95% confidence intervals (CIs) and interquartile ranges. The optimal cutoff levels of drain amylase on PODs 1, 2, and 3, and serum and drain CRP levels on PODs 2, 3, and 4 for differentiating the CR-POPF group from the non-CR-POPF group were determined by constructing ROC curves. The curves were generated by calculating the sensitivities and specificities of these levels at several predetermined cutoffs. Logistic regression analysis and ROC analysis were performed to identify the predictive factors for CR-POPFs. Model calibration was assessed using the Hosmer–Lemeshow goodness-of-fit test (10 deciles, *p*-values >0.05 indicating good calibration). In addition, calibration performance was evaluated using a calibration belt with 95% CIs, which compares the agreement between predicted and observed probabilities of CR-POPF. Each variable’s odds ratio (OR) was reported with a 95% CI. Statistical significance was set at two-tailed *p*-values <0.05. All statistical analyses were conducted using the JMP statistical software (version 17.0; SAS Institute, Cary, NC, USA).

## Results

### Patient Characteristics and Postoperative Outcomes

Among the 388 initially enrolled patients who underwent PD with curative intent, four patients with missing data were excluded; the remaining 384 patients were included in this study. The median patient age was 71 years (interquartile range 62–77). The most common histopathological diagnoses included pancreatic ductal adenocarcinoma (52.3%), ampullary carcinoma (10.9%), intraductal papillary mucinous neoplasm (10.9%), distal cholangiocarcinoma (9.6%), and neuroendocrine neoplasm (6.3%). The overall morbidity (grade ≥III) and relaparotomy rates were 17.7% and 2.3%, respectively. CR-POPF occurred in 58 patients (15.1%), including grade B and C POPF in 50 (13.0%) and eight (2.1%) patients, respectively. Grade B/C DGE, PPH, bile leakage, and POH occurred in 16 (4.2%), 15 (3.9%), 20 (5.2%), and 119 (30.7%) patients, respectively. The 30-day mortality rate was 0.5%. The patient characteristics and operative outcomes are summarized in Table 1.

**Table 1:** Patient characteristics and postoperative outcomes

Characteristics and factors	n=384
Age (years)	71 (62–77)
Sex, male/female	238/146
BMI (kg/m^2^)	21.4 (19.6–23.7)
*Preoperative patient characteristics*	
Diabetes	126 (32.8)
Preoperative bile drainage	177 (46.1)
Neoadjuvant chemotherapy	90 (23.4)
*Diagnosis*	
Pancreatic ductal adenocarcinoma	201 (52.3)
Distal cholangiocarcinoma	37 (9.6)
Ampullary carcinoma	42 (10.9)
Gallbladder carcinoma	5 (1.3)
IPMN	42 (10.9)
NEN	24 (6.3)
SPN	3 (0.8)
Chronic pancreatitis	18 (4.7)
Others	12 (3.1)
*ASA-PS classification*	
I	28 (7.3)
II	332 (86.5)
III	24 (6.3)
*Major complications*	
Morbidity grade ≥III	68 (17.7)
Relaparotomy	9 (2.3)
*POPF*	
Biochemical fistula	90 (23.4)
Grade B	50 (13.0)
Grade C	8 (2.1)
DGE grade B/C	16 (4.2)
PPH grade B/C	15 (3.9)
Bile leakage	20 (5.2)
Abdominal abscess	4 (1.0)
Anastomotic leakage	6 (1.6)
Ascites	6 (1.6)
Ileus	3 (0.8)
Others	14 (3.6)
POH	119 (30.7)
Postoperative 30-day mortality	2 (0.5)
Hospital stay, days	20 (16–28)

Data are presented as median (interquartile range) or n (%) unless otherwise indicated

*ASA-PS* American Society of Anesthesiologists physical status; *BMI* Body mass index; *DGE* Delayed gastric emptying; *IPMN* Intraductal papillary mucinous neoplasm; *IQR* Interquartile range; *nen* neuroendocrine neoplasm; *POH* Postoperative hyperamylasemia; *POPF* Postoperative pancreatic fistula; *PPH* Postpancreatectomy hemorrhage; *SPN* Solid-pseudopapillary neoplasm

### Operative Procedure and Findings

Compared with the non-CR-POPF group, the CR-POPF group included more male patients (81.0% vs. 58.6%; *p*=0.001), fewer patients with pancreatic cancer (25.9% vs. 57.1%; *p*<0.001), more patients with a soft pancreatic texture (84.5% vs. 45.1%; *p*<0.001), and more patients with a small pancreatic duct (diameter ≤3 mm; 74.1% vs. 40.5%; *p*<0.001; Table 2). Other variables, such as age ≥70 years, body mass index ≥25 kg/m^2^, diabetes, preoperative biliary drainage, neoadjuvant chemotherapy, ASA-PS classification, pancreatic anastomosis after resection (pancreaticogastrostomy or pancreaticojejunostomy), portal vein/superior mesenteric vein resection, arterial resection, estimated blood loss ≥600 mL, intraoperative blood cell transfusion, and POH, were not significantly different between the two groups.

**Table 2:** Patient characteristics and perioperative outcomes according to clinically relevant postoperative pancreatic fistula (CR-POPF)

Variables	All patients (n=384)	Non-CR-POPF (n=326)	CR-POPF (n=58)	*p*-Value
Age ≥70 years	208 (54.2)	173 (53.1)	35 (60.3)	0.305
Sex, male	238 (62.0)	191 (58.6)	47 (81.0)	0.001
BMI ≥25 kg/m^2^	59 (15.6)	48 (15.0)	11 (19.0)	0.438
Diabetes	126 (32.8)	110 (33.7)	16 (27.6)	0.358
Preoperative bile drainage	177 (46.1)	155 (47.6)	22 (37.9)	0.176
Neoadjuvant chemotherapy	90 (23.4)	79 (24.2)	11 (19.0)	0.383
ASA-PS 1/2/3	28/332/24	26/279/21	2/53/3	0.427
Pancreatic anastomosis, PG/PJ	367/17	310/16	57/1	0.277
PV/SMV resection	82 (21.4)	75 (23.0)	7 (12.1)	0.061
Arterial resection	21 (5.5)	19 (5.8)	2 (3.5)	0.463
Pancreatic cancer	201 (52.3)	186 (57.1)	15 (25.9)	<0.001
Pancreatic texture soft	196 (51.0)	147 (45.1)	49 (84.5)	<0.001
Pancreatic duct diameter ≤3 mm	175 (45.6)	132 (40.5)	43 (74.1)	<0.001
Estimated blood loss ≥600 mL	143 (37.2)	121 (37.1)	22 (37.9)	0.906
Blood cell transfusion	48 (12.5)	43 (13.2)	5 (8.6)	0.332
POH	119 (30.7)	95 (28.9)	24 (40.7)	0.070

Data are presented as n (%) unless otherwise indicated

*ASA-PS* American Society of Anesthesiologists physical status; *BMI* Body mass index; *CR-POPF* Clinically relevant postoperative pancreatic fistula; *PG* Pancreaticogastrostomy; *PJ* Pancreaticojejunostomy; *PPOH* Postoperative hyperamylasemia; *V/SMV* Portal/superior mesenteric vein

### Postoperative Laboratory Data According to CR-POPF Status and the Correlation Between Serum and Drain CRP Levels

**Fig. 1:**
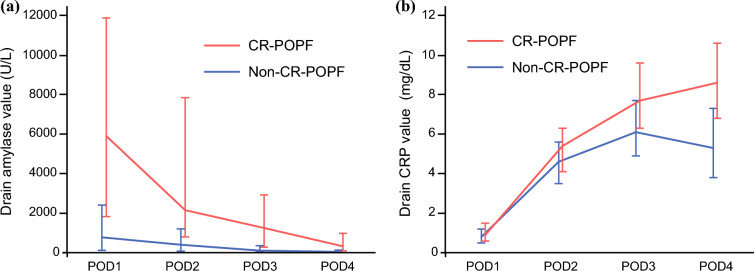
Temporal trends of (**a**) drain amylase and (**b**) C-reactive protein (CRP) levels from postoperative day (POD) 1 to POD 4 in patients with and without clinically relevant postoperative pancreatic fistula (CR-POPF). Data are presented as medians with interquartile ranges

**Fig. 2:**
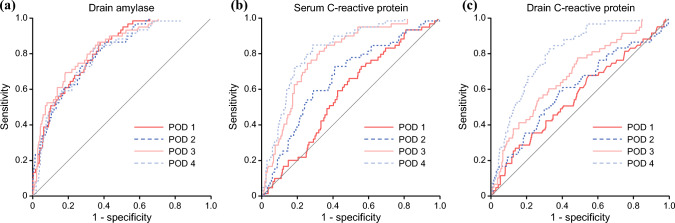
Receiver operating characteristic curves of the predictors of clinically relevant postoperative pancreatic fistulas (grades B/C). **a** Drain amylase, **b** serum C-reactive protein, and **c** drain C-reactive protein levels on postoperative days (PODs) 1–4

**Fig. 3:**
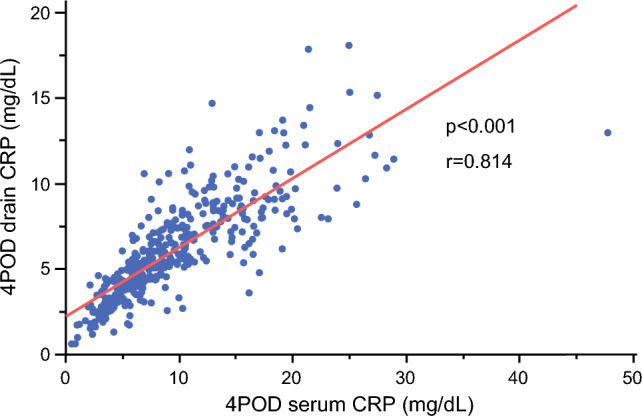
Correlation between serum and drain C-reactive protein (CRP) levels on postoperative day (POD) 4. Statistical analyses were performed using correlation analysis, and statistical significance was determined at *p*<0.05

**Fig. 4:**
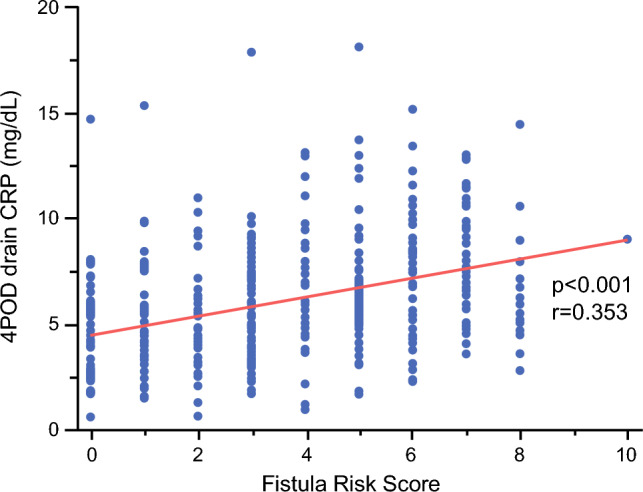
Correlation between the Fistula Risk Score and drain C-reactive protein (CRP) levels on postoperative day (POD) 4. Statistical analyses were performed using correlation analysis, and statistical significance was determined at *p*<0.05

The temporal trends of drain CRP and amylase levels according to CR-POPF status are shown in Fig. 1. Both markers were higher in patients who developed CR-POPF than in those without CR-POPF throughout the postoperative course. Univariate analysis revealed that drain amylase levels on PODs 1, 2, and 3, and serum and drain CRP levels on PODs 2, 3, and 4 were significantly associated with CR-POPF (Table 3). No drains were removed by POD 3. ROC analysis of individual predictive factors for CR-POPF with significant differences demonstrated that the area under the curve values of drain amylase were 0.815, 0.800, and 0.824 on PODs 1, 2, and 3, respectively; those of serum CRP were 0.660, 0.786, and 0.820 on PODs 2, 3, and 4, respectively; and those of drain CRP were 0.605, 0.684, and 0.803 on PODs 2, 3, and 4, respectively (Fig. 2). Therefore, drain amylase levels on POD 3 and serum and drain CRP levels on POD 4 were identified as the most accurate predictors of CR-POPF. The optimal cutoff values derived from the ROC curve for drain amylase and serum and drain CRP were 523 U/L, 6.5 mg/dL, and 10.4 mg/dL, respectively. Additionally, drain CRP level on POD 4 exhibited a strong positive correlation with serum CRP level on POD 4 (Fig. 3; *p*<0.001, *r*=0.814) and a significant positive correlation with the FRS (Fig. 4; *p*<0.001, *r*=0.353), indicating alignment between postoperative inflammatory response and pre-/intraoperative risk assessment.

**Table 3:** Postoperative laboratory data according to clinically relevant postoperative pancreatic fistula (CR-POPF) using the area under the receiver operating characteristics curve (AUC)

Variables	All patients (n=384)	Non-CR-POPF (n=326)	CR-POPF (n=58)	*p*-Value	AUC
*Drain amylase (U/L)*					
POD 1	1123 (182–3272)	778 (120–2408)	5902 (1828–11877)	<0.001	0.815
POD 2	530 (108–1539)	409 (80–1207)	2147 (802–7842)	<0.001	0.800
POD 3	137 (43–550)	104 (33–358)	1246 (280–2926)	<0.001	0.824
POD 4	57 (21–180)	45 (19–133)	326 (92–980)	0.585	
*Serum CRP (mg/dL)*					
POD 1	8.6 (6.9–10.6)	8.5 (6.9–10.6)	9.4 (7.7–10.5)	0.304	
POD 2	16.8 (12.8–20.3)	16.2 (12.6–19.6)	19.7 (16.5–22.8)	<0.001	0.660
POD 3	13.9 (9.4–19.1)	12.9 (8.8–17.5)	19.6 (16.4–23.1)	<0.001	0.786
POD 4	8.5 (5.8–13.1)	7.7 (5.3–10.8)	15.0 (11.2–18.4)	<0.001	0.820
*Drain CRP (mg/dL)*					
POD 1	0.8 (0.5–1.2)	0.8 (0.5–1.2)	0.9 (0.6–1.5)	0.880	
POD 2	4.7 (3.5–5.7)	4.6 (3.5–5.6)	5.4 (4.1–6.3)	0.008	0.605
POD 3	6.4 (5.0–8.0)	6.1 (4.9–7.7)	7.7 (6.3–9.6)	<0.001	0.684
POD 4	5.7 (4.1–8.0)	5.3 (3.8–7.3)	8.6 (6.8–10.6)	<0.001	0.803

Data are presented as median (interquartile range)

*CRP* C-reactive protein; *POD* Postoperative day

### Risk Factors for CR-POPF

Using univariate analysis, male sex, nonpancreatic cancer, soft pancreatic texture, pancreatic duct diameter ≤3 mm, drain amylase level ≥523 U/L on POD 3, serum CRP level ≥10.4 mg/dL on POD 4, and drain CRP level ≥6.5 mg/dL on POD 4 were identified as risk factors for CR-POPF (Table 4). In the multivariate analysis, serum CRP level on POD 4 was excluded because of the strong confounding effect of drain CRP level on the same POD. Multivariate logistic regression analysis using these variables revealed that a pancreatic duct diameter ≤3 mm (OR 2.63; 95% CI 1.24–5.57; *p*=0.012), drain amylase level ≥523 U/L on POD 3 (OR 3.83; 95% CI 1.84–7.96;* p*<0.001), and drain CRP level ≥6.5 mg/dL on POD 4 (OR 4.95; 95% CI 2.27–10.81; *p*<0.001) were significant risk factors for CR-POPF (Table 4). The Hosmer–Lemeshow test indicated good calibration of the model. The calibration belt analysis also demonstrated no significant deviation between predicted and observed probabilities, confirming adequate calibration of the model. The respective sensitivity, specificity, positive predictive value (PPV), and negative predictive value (NPV) of the factors were 74.1%, 59.5%, 24.6%, and 92.8% for a pancreatic duct diameter ≤3 mm; 69.0%, 81.3%, 39.6%, and 93.6% for a drain amylase level ≥523 U/L on POD 3; 84.5%, 71.2%, 34.3%, and 96.3% for a serum CRP level ≥10.4 mg/dL on POD 4; and 82.8%, 67.2%, 31.0%, and 95.6% for a drain CRP level ≥6.5 mg/dL on POD 4 (Table 5).

**Table 4:** Risk factors for clinically relevant postoperative pancreatic fistula (CR-POPF) in the univariate and multivariate analyses

Variables	Univariate analyses			Multivariate analyses	
	Non-CR-POPF (n=326)	CR-POPF (n=58)	*p*-value	OR (95% CI)	*p*-value
Male sex	191 (58.6)	47 (81.0)	0.001	2.12 (0.96–4.69)	0.062
Nonpancreatic cancer	140 (42.9)	43 (74.1)	<0.001	1.39 (0.65–2.98)	0.390
Soft pancreatic texture	147 (45.1)	49 (84.5)	<0.001	1.35 (0.52–3.48)	0.538
Pancreatic duct diameter ≤3 mm	132 (40.5)	43 (74.1)	<0.001	2.63 (1.24–5.57)	0.012
Drain amylase on POD 3 ≥523 U/L	61 (18.7)	40 (69.0)	<0.001	3.83 (1.84–7.96)	<0.001
Drain CRP on POD 4 ≥6.5 mg/dL	107 (32.8)	48 (82.8)	<0.001	4.95 (2.27–10.81)	<0.001

Data are presented as n (%) unless otherwise indicated

*CI* Confidence interval; *CRP* C-reactive protein; *OR* Odds ratio; *POD* Postoperative day; *PV/SMV* Portal/superior mesenteric vein

**Table 5:** Sensitivity, specificity, positive predictive value, and negative predictive value of risk factors for clinically relevant postoperative pancreatic fistula

Variables	Sensitivity (%)	Specificity (%)	Positive predictive value (%)	Negative predictive value (%)
Pancreatic duct diameter ≤3 mm	74.1	59.5	24.6	92.8
Drain amylase on POD 3 ≥523 U/L	69.0	81.3	39.6	93.6
Serum CRP on POD 4 ≥10.4 mg/dL	84.5	71.2	34.3	96.3
Drain CRP on POD 4 ≥6.5 mg/dL	82.8	67.2	31.0	95.6

Data are presented as percentages

*CRP* C-reactive protein; *POD* Postoperative day

## Discussion

In this study, we demonstrated that drain CRP level on POD 4 is an independent predictive factor for CR-POPF after PD that exhibits a strong positive correlation with serum CRP level on POD 4.

CR-POPF is a common complication of pancreatectomy and a major cause of postoperative morbidity and mortality.^2,5,33^ The reported incidence of CR-POPF after pancreatic resection ranges from 3% to 45%;^4,34^ however, an incidence of 15.2% was observed. Baseline characteristics apart from sex, such as age, preoperative biliary drainage, neoadjuvant chemotherapy, and ASA-PS classification in the CR-POPF group were not significantly different from those in the non-CR-POPF group. These results were consistent with other studies.^35,36^ Estimated blood loss was not significantly associated with CR-POPF in this study. This is consistent with recent evidence indicating that the predictive value of blood loss is lower than that of pancreatic duct diameter and gland texture.^8^ As newer risk models no longer include blood loss as a key factor, its impact appears limited, particularly in our relatively homogeneous cohort with standardized surgical techniques that minimized variability. This study found that a pancreatic duct diameter ≤3 mm is a risk factor for POPF, consistent with previous findings on surgery-related risk factors for POPF.^7,8^

Drain CRP level on POD 4 was identified as an independent predictive factor for CR-POPF after PD. Previous studies have reported that the measurement of drain CRP level can be used to detect anastomotic leakage in patients undergoing colorectal resection.^37,38^ Moreover, serum CRP level on POD 4 is associated with CR-POPF.^6,16,20,21^ In this study, drain CRP level on POD 4 exhibited a strong positive correlation with serum CRP level on POD 4; therefore, it is plausible that drain CRP level is also associated with CR-POPF. Therefore, the use of drain CRP level may be advantageous over serum CRP for predicting CR-POPF as it is noninvasive and requires no blood tests.

A previous study reported that CRP levels in ascitic fluid increase due to intra-abdominal inflammation in malignancy.^39,40^ Studies have also shown that CRP levels in pleural fluid may reflect serum CRP levels due to the increased diffusion of CRP from blood because of inflammation-associated capillary leakage.^41^ Hence, it is plausible that a positive correlation exists between serum and drain CRP levels through a similar mechanism.

Notably, drain CRP level on POD 4, but not on PODs 1 or 3, is the most accurate predictor of CR-POPFs. Reportedly, inflammatory responses triggered by pancreatic surgery occur postoperatively, and data on inflammatory markers can be obtained after recovery.^6,20^ Serum inflammatory markers (e.g., CRP levels) typically peak on POD 3 and decrease gradually thereafter, following a pattern similar to that observed in drain fluid. However, in patients with CR-POPFs, drain CRP levels may not decrease because of the occurrence of CR-POPFs. While amylase indicates pancreatic juice leakage, CRP in drain fluid may reflect the inflammatory burden associated with clinically relevant fistula, and its delayed peak on POD 4 may help confirm the clinical severity of POPF.

Notably, the NPV of drain CRP level on POD 4 was significantly high (95.6%). As an alternative to serum CRP levels, a high NPV is important for safe and appropriate drain management. Studies have shown a relationship between postoperative clinical data and CR-POPFs following PD. Kawai et al.^42^ reported that a combination of the two factors – a serum albumin level ≤3.0 g/dL and leukocyte count >9800 mm^3^ on POD 4 – is predictive of CR-POPFs (PPV=88%, NPV=85%). Molinari et al.^9^ reported that a drain amylase level >5000 U/L on POD 1 was the only significant predictive factor of POPF development (PPV=48%, NPV=98%). Conversely, our study introduced that the use of drain CRP on POD 4 would be appropriate for safe drain removal. Drain CRP is a non-invasive method for patients with surgical drains already in place and may reduce unnecessary blood sampling. Although serum CRP showed similar area under the curve values, the high NPV of drain CRP (95.6%) may facilitate safe early drain removal without additional interventions. In addition, drain CRP showed a significant positive correlation with the original FRS by Callery et al.^7^, indicating that postoperative inflammatory changes are aligned with pre- and intraoperative risk assessments. Importantly, the modest strength of this correlation suggests that drain CRP provides complementary postoperative information beyond existing risk stratification models. Thus, combining preoperative risk assessment with postoperative drain CRP monitoring may improve the accuracy and clinical utility of early CR-POPF prediction.

This study had some limitations that should be considered when interpreting the results. First, this was a retrospective, single-center study with a relatively small cohort; therefore, our results are vulnerable to potential bias, despite including the largest number of cases to date. Second, the occurrence of POPF in patients with mildly elevated CRP levels may be due to a lack of drain removal, as suggested by Bassi et al*.*^10^ Third, data on drain CRP levels after day 5 could not have been collected if the drain had been removed early. Fourth, post-pancreatectomy acute pancreatitis could not be evaluated because the necessary biochemical and imaging data were not available in our dataset. Further randomized controlled studies involving a larger number of patients, with systematic data collection including post-pancreatectomy acute pancreatitis, are required to confirm our study findings.

In conclusion, the present study indicates that drain CRP level on POD 4 is a strong predictor of CR-POPF, suggesting that drain CRP levels on POD 4 may be an alternative criterion to serum CRP level following PD. This possibility opens new frontiers in postoperative management because blood sampling is not required. Patients not at risk (NPV=95.6% for all pancreatic resection procedures) may be candidates for earlier drain removal without the need for blood sampling, thus avoiding the risk of infections and minimizing invasiveness.
